# Financial costs of assisted reproductive technology for patients in low- and middle-income countries: a systematic review

**DOI:** 10.1093/hropen/hoad007

**Published:** 2023-03-01

**Authors:** Purity Njagi, Wim Groot, Jelena Arsenijevic, Silke Dyer, Gitau Mburu, James Kiarie

**Affiliations:** Maastricht Graduate School of Governance, United Nations University-MERIT, Maastricht University, Maastricht, The Netherlands; Maastricht Graduate School of Governance, United Nations University-MERIT, Maastricht University, Maastricht, The Netherlands; Department of Health Services Research, Faculty of Health, Medicine and Life Sciences, Maastricht University, Maastricht, The Netherlands; School of Governance, Faculty of Law, Economics and Governance, Utrecht University, Utrecht, The Netherlands; Department of Obstetrics and Gynaecology, University of Cape Town, Cape Town, South Africa; UNDP-UNFPA-UNICEF-WHO-World Bank Special Programme of Research, Development and Research Training in Human Reproduction (HRP), Department of Sexual and Reproductive Health and Research (SRH), World Health Organization, Genève, Switzerland

**Keywords:** assisted reproductive technology, in vitro fertilization, infertility, medical costs, out of pocket, systematic review, low- and middle-income countries

## Abstract

**STUDY QUESTION:**

What are the direct costs of assisted reproductive technology (ART), and how affordable is it for patients in low- and middle-income countries (LMICS)?

**SUMMARY ANSWER:**

Direct medical costs paid by patients for infertility treatment are significantly higher than annual average income and GDP per capita, pointing to unaffordability and the risk of catastrophic expenditure for those in need.

**WHAT IS KNOWN ALREADY:**

Infertility treatment is largely inaccessible to many people in LMICs. Our analysis shows that no study in LMICs has previously compared ART medical costs across countries in international dollar terms (US$PPP) or correlated the medical costs with economic indicators, financing mechanisms, and policy regulations. Previous systematic reviews on costs have been limited to high-income countries while those in LMICs have only focussed on descriptive analyses of these costs.

**STUDY DESIGN, SIZE, DURATION:**

Guided by the preferred reporting items for systematic reviews and meta-analyses (PRISMA), we searched PubMed, Web of Science, Cumulative Index of Nursing and Allied Health Literature, EconLit, PsycINFO, Latin American & Caribbean Health Sciences Literature, and grey literature for studies published in all languages from LMICs between 2001 and 2020.

**PARTICIPANTS/MATERIALS, SETTING, METHODS:**

The primary outcome of interest was direct medical costs paid by patients for one ART cycle. To gauge ART affordability, direct medical costs were correlated with the GDP per capita or average income of respective countries. ART regulations and public financing mechanisms were analyzed to provide information on the healthcare contexts in the countries. The quality of included studies was assessed using the Integrated Quality Criteria for Review of Multiple Study designs.

**MAIN RESULTS AND THE ROLE OF CHANCE:**

Of the 4062 studies identified, 26 studies from 17 countries met the inclusion criteria. There were wide disparities across countries in the direct medical costs paid by patients for ART ranging from USD2109 to USD18 592. Relative ART costs and GDP per capita showed a negative correlation, with the costs in Africa and South-East Asia being on average up to 200% of the GDP per capita. Lower relative costs in the Americas and the Eastern Mediterranean regions were associated with the presence of ART regulations and government financing mechanisms.

**LIMITATIONS, REASONS FOR CAUTION:**

Several included studies were not primarily designed to examine the cost of ART and thus lacked comprehensive details of the costs. However, a sensitivity analysis showed that exclusion of studies with below the minimum quality score did not change the conclusions on the outcome of interest.

**WIDER IMPLICATIONS OF THE FINDINGS:**

Governments in LMICs should devise appropriate ART regulatory policies and implement effective mechanisms for public financing of fertility care to improve equity in access. The findings of this review should inform advocacy for ART regulatory frameworks in LMICs and the integration of infertility treatment as an essential service under universal health coverage.

**STUDY FUNDING/COMPETING INTEREST(S):**

This work received funding from the UNDP-UNFPA-UNICEF-WHO-World Bank Special Programme of Research, Development and Research Training in Human Reproduction (HRP), a cosponsored programme executed by the World Health Organization (WHO). The authors declare no competing interests.

**TRIAL REGISTRATION NUMBER:**

This review is registered with PROSPERO, CRD42020199312.

**WHAT DOES THIS MEAN FOR PATIENTS?**

This review appraises the literature on the costs of assisted reproductive technology (ART) borne by individuals, its affordability, and the association with government financing and ART regulations, between 2001 and 2020.

To assess affordability, we examined the correlation of the direct medical costs paid by patients for one ART cycle with the respective countries' GDP per capita or average income.

In conclusion, based on the findings, there were significant inequities in access to ART, and many patients in LMICs are still unable to afford it due to prohibitive costs. Better policies and government financial mechanisms are needed to improve affordability for patients in LMICs.

## Introduction

Infertility is a disease defined as the failure to achieve a clinical pregnancy after 12 months or more of regular unprotected sexual intercourse (Zegers-Hochschild *et al.*, 2017; WHO, 2019). While reported prevalence estimates vary widely due to different methodologies, global estimates show that between 48.5 and 72.4 million couples have infertility (Boivin *et al.*, 2007; Mascarenhas *et al.*, 2012). The prevalence of infertility among reproductive-aged couples ranges between 12.6% and 17.5% worldwide, with relatively higher prevalence rates in some regions such as the Americas, Western Pacific, African, and European regions (Cox et al., 2022). Regional disparities in prevalence reflect differences in sexual and reproductive health and rights and differences in access to and quality of health care, which, in turn, are further influenced by environmental, cultural, and societal factors (Ombelet, 2011).

Assisted reproductive technology (ART) for the treatment of infertile couples (or persons) is considered an important biomedical intervention throughout the world (Bitler and Schmidt, 2012; Inhorn and Patrizio, 2015; Sharma *et al.*, 2018). However, there are marked disparities in the availability, quality, and delivery of infertility care services between high-income countries (HICs) and low- and middle-income countries (LMICs) (Nachtigall, 2006). Even though ART has existed for over four decades, it remains either unavailable or inaccessible to most people in resource-poor settings (Sharma *et al.*, 2009; Allahbadia, 2013; Botha *et al.*, 2018; Ombelet and Onofre, 2019). Apart from being costly, ART is also often time-consuming, physically and emotionally strenuous, and without certainty about its outcome (Domar *et al.*, 2012; Rouchou, 2013). Moreover, in many resource-limited settings, such as sub-Saharan Africa, infertility is often neglected due to many competing health needs, as well as the relatively high fertility rates and large family sizes, which may not only mask infertility in populations (Asemota and Klatsky, 2015), but may even have created disincentives to public funding of infertility treatment. As a result, in many LMICs, government-funded infertility treatments are either limited or non-existent and are excluded from health insurance packages (Adewumi, 2017), despite the associated high costs to patients (Ombelet, 2011; Insogna and Ginsburg, 2018). Governments’ insufficient capacity or commitment to respond to infertility means that many couples pay for their treatment out of pocket (OOP), making cost an important barrier to access (Roa-Meggo, 2012; Casebolt, 2020), likely resulting in treatment inequalities (Dyer *et al.*, 2013). Furthermore, even in HICs, the level of access to ART treatments is reported to be sensitive to the costs paid by patients (Chambers *et al.*, 2014).

This means that in LMICs, ART can generally only be accessed by the well-off, paying OOP via predominantly private health facilities (Hall and Hanekom, 2020). Nonetheless, the desire for a child often encourages couples to make significant financial sacrifices and even suffer catastrophic financial hardship to obtain infertility care (Dyer and Patel, 2012; Dyer *et al.*, 2013). Moreover, the willingness and financial ability to undergo more than one ART cycle often depend on the OOP payments incurred (Wu, 2019).

It is possible that costs may vary across countries based on economic parameters, laws, regulations, and insurance coverage for assisted reproduction (Klitzman, 2017). Therefore, a better understanding of the economic implications of infertility is needed to inform policies supporting equitable access to ART without undue financial risks to patients in LMICs. This is particularly relevant given that a sustainable establishment of infertility services in developing countries depends on models that involve treatment financing by governments (Sallam, 2008). This review, therefore, aims to provide new evidence on financial OOP costs for ART through a rigorous systematic review by including all languages, citing conversion of costs into international USD and using purchasing power parity to facilitate comparison across countries and regions, evaluating affordability by drawing on GDP per capita or average national income, and assessing the relationship between cost burdens and local ART policies and financing mechanisms.

## Materials and methods

In keeping with the study protocol published in the PROSPERO database (CRD42020199312) and elsewhere (Njagi *et al.*, 2020), this study was conducted according to the preferred reporting items for systematic reviews and meta-analyses (PRISMA) statement (Moher *et al.*, 2009).

### Search strategy and selection criteria

We searched articles indexed in the following databases: PubMed, Cumulative Index of Nursing and Allied Health Literature (CINAHL), Web of Science, EconLit, PsycINFO, and Latin American & Caribbean Health Sciences Literature (LILACS). The search of databases was complemented by a search of the grey literature from Google Scholar and online libraries of relevant organizations such as the World Health Organization (WHO), the International Federation of Gynaecology and Obstetrics (FIGO), the International Federation of Fertility Societies (IFFS), and the International Committee for Monitoring Assisted Reproductive Technologies (ICMART). Proceedings and abstracts from the following conferences were also searched: ESHRE, the American Society for Reproductive Medicine (ASRM), the Latin American Network of Assisted Reproduction (REDLARA), and the Asia Pacific Initiative on Reproduction (ASPIRE).

In addition, we conducted a forward and backward reference search of authors mentioned in selected articles.

Search terms included ‘reproductive techniques, assisted’ [MeSH Terms] OR ‘fertilization in vitro’ [MeSH Terms] OR ‘insemination, artificial’ [MeSH Terms] OR ‘infertility’ AND ‘Costs’ [MeSH Terms] OR ‘health expenditures’ [MeSH Terms] OR ‘fee for service’ [MeSH Terms] OR ‘Out-of-pocket’ OR ‘Payments’ AND ‘developing countries’ [MeSH Terms] OR ‘Low-income countries’ OR ‘Middle-income countries’. A detailed search strategy for PubMed is shown in Supplementary Table SI.

### Inclusion criteria

The search was restricted to studies published between 2001 and 2020 and included articles in all languages, irrespective of their study designs. In addition, studies were only included if they: (i) were undertaken in LMICs, as defined by the World Bank (World Bank, 2021a) and (ii) reported the direct medical or non-medical costs of ART incurred by patients. These were categorized into both primary and secondary outcomes of interest, respectively.

### Definition of outcome and analysis parameters

The following parameters and definitions were used in the analysis of included studies.

Direct medical costs: medical costs paid to ART health providers by patients including pre-ART work-up, consultation, drugs, and procedural and laboratory costs.

Direct non-medical costs: non-medical costs incurred by patients such as transport, accommodation, and food.

ART regulation: the presence of legal policies, laws, or regulations related to ART practice.

ART financing: the presence of government mechanism of ART funding.

Regions: the classification of the world into WHO regions for purposes of administration and reporting.

### Quality assessment

All studies eligible for full review were assessed by two reviewers using the Integrated Quality Criteria for Review of Multiple Study designs (ICROMS) tool (Zingg *et al.*, 2016). ICROMS incorporates existing quality assessment criteria of various study designs (randomized, controlled before-and-after, and interrupted time series, non-controlled before-and-after studies, cohort studies, and qualitative studies) consisting of a ‘decision matrix’ and a list of quality standards unique to each study design using a scoring system. The ‘decision matrix’ establishes the robustness of the study based on two factors: a mandatory criterion that considers some of the quality elements as mandatory to be met and a minimum score requirement for each study type that equates to 60% of the maximum total points attributable to a specific study design to guarantee both relevance and methodological rigor (Zingg *et al.*, 2016). For cross-sectional studies, we applied the criteria for non-controlled studies complemented by the quality assessment tool for observational and cross-sectional studies provided by the National Institutes of Health (Downes *et al.*, 2016; Ma *et al.*, 2020).

### Data extraction and analysis

#### Extraction of study characteristics

A standardized matrix was prepared for data extraction. This included the title, year of publication, the year the study was undertaken, country and region of study, study design, sample size, and the target population. Two reviewers extracted data. Differences were resolved through discussion, while a third reviewer examined the outputs for consistency.

#### Extraction of primary and secondary outcome data

We extracted and computed the total direct medical and non-medical costs for one ART cycle from the reported data. Where several direct medical costs for various services rendered were reported, we calculated the total direct medical cost for one cycle. For studies documenting the total medical costs for all study participants per ART cycle, we divided the total medical costs by the total number of participants. From studies that provided the range of minimum and maximum direct costs, we extracted the average.

#### Extraction of macro-economic data

To complement the primary and secondary outcome data, we used data on country-specific indicators, including GDP per capita as a proxy of income and well-being (Boarini *et al.*, 2006; Ke and Saksena, 2011). Because access is determined by income, we analyzed average income from the World Bank's PovcalNet for countries, where data were available, to estimate the share of the population for which infertility treatment is inaccessible. Estimates of average income are based on the population's income distribution and the percentage poverty headcount (World Bank, 2021b).

All calculated costs were converted from local currencies into US dollars (LCU per US$, period average) applying exchange rates applicable to the year the study was conducted or published when the year of study was not reported. For the evaluation of affordability, the direct costs were calculated as a percentage of GDP per capita. To facilitate cross-country comparisons, we further adjusted the direct costs and GDP per capita to the international dollar using the purchasing power parity (PPP) conversion rate (World Bank, 2021c). This conversion helps to equalize the medical costs and draw cross-country comparison while eliminating price level differences (Boarini *et al.*, 2006).

#### Extraction of public financing and regulatory data

Applicable data on both the regulations and government financing of ART were extracted from the International Federation of Fertility Societies’ Surveillance (IFFS) (2019).

## Results

### Study selection

The initial search identified 4062 studies. After removing 175 duplicates, the remaining studies were screened, and 3812 were excluded based on title and abstracts. The abstracts of studies written in languages other than English were translated using Google Translate. If eligible for inclusion, full translation was conducted using an online document translator (www.onlinedoctranslator.com). The translated versions were compared with the Google Translate version and assessed for consistency by two reviewers.

**Figure 1.**
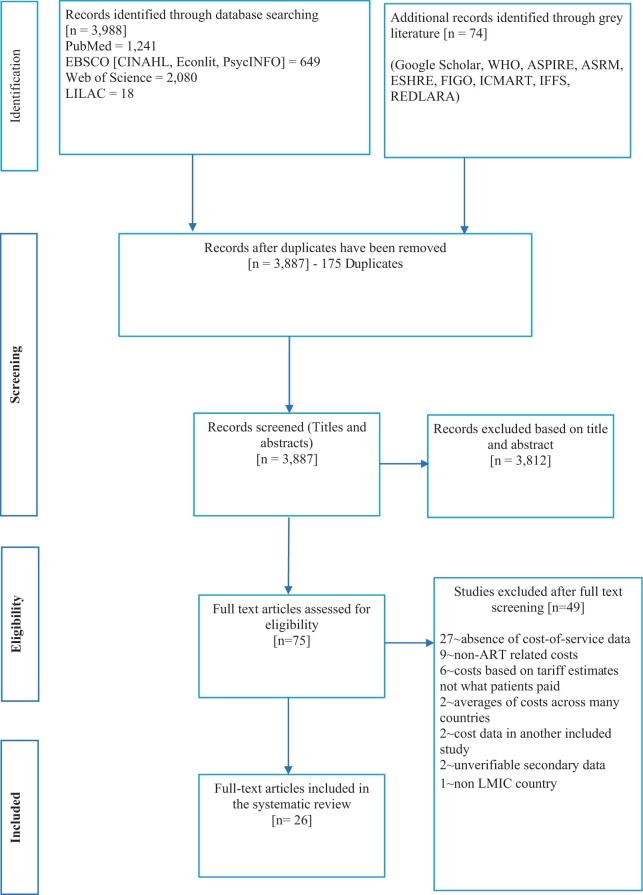
**Preferred reporting items for systematic reviews and meta-analyses (PRISMA) flow chart.** Data flow through the different phases of studies selection, showing the number of records identified, screened for eligibility, included and excluded, and the reasons for exclusions. LMIC, low- and middle-income countries.

As a result, 75 studies were screened in full text, and 49 of these studies did not meet the inclusion criteria mainly due to the absence of cost-of-service data. Also excluded were economic evaluations that derived the cost of ART based on tariff estimates rather than what patients paid, studies that provided non-ART treatment costs, reviews that provided averages across countries, and some qualitative reviews citing unverifiable secondary data. Ultimately, 26 studies met the inclusion criteria for this review. The PRISMA flow chart (Fig. 1) summarizes the study selection process.

### Quality assessment and risk of bias

Using the ICROMS tool, included studies were classified according to their design and quality criteria. The risk of bias was low/moderate across the studies, given that more than half of the included studies scored above the minimum score (Table I). The detailed scores per study are presented in Supplementary Table SII.

**Table I:** Quality assessment of included studies (i.e. minimum expected ICROMS score).

	Studies above (>) minimum score		Studies below (<) minimum score			
Study design	Total (N)	Percent (%)	Total (N)	Percent (%)	Total studies	Minimum score for study design
Controlled before–after (CBA)	1	NA	NA	NA	1	18
Non-controlled before–after (NCBA)	8	66.7%	4	33.3%	12	22
Qualitative studies	5	38.5%	8	61.5%	13	16
**Total**	**14**		**12**		**26**	

ICROMS, Integrated Quality Criteria for Review of Multiple Study designs.

One controlled cost-effectiveness study was assessed using the controlled before–after (CBA) criterion and scored 17 out of a minimum score of 18. Twelve studies were assessed using the non-controlled before–after (NCBA) criterion (minimum score of 22). Of these, eight studies scored above the minimum score, while four studies were slightly below. Thirteen qualitative studies were assessed using the relevant criteria with an expected minimum score of 16. Of these, five studies scored above the minimum score while eight scored below. Of the eight, one scored slightly below the minimum score, while the other seven were qualitative reviews and not primary studies. The studies that scored below the minimum quality scores were mainly qualitative studies and reviews because they did not report strategies for managing bias in sampling and reporting. However, these studies still provided relevant data related to the outcome of interest of this review.

### Sensitivity analysis on risk of bias

In addition, we further conducted sensitivity analysis based on the type of studies and the cost of ART. There was no statistical difference in the means of the reported ART costs between studies that scored less (<) or higher (>) than the minimum score for both quantitative and qualitative studies. The sensitivity analyses show that excluding studies with below minimum quality scores did not impact the outcome of interest. Furthermore, those that did not meet the minimum scores were qualitative studies mainly from Africa, where primary quantitative studies on the cost of infertility were limited.

### Characteristics of the included studies

There were 13 quantitative and 13 qualitative studies. The former included cost-effectiveness, cross-sectional, mixed-methods and prospective studies. Of the qualitative studies, six were primary, while seven were descriptive reviews. The latter were included as they provided relevant information on the primary outcome measure. Geographically, the studies covered a total of 17 countries spread across five WHO regions, including the African region (n = 9), Eastern Mediterranean Region (n = 5), Region of Americas (n = 5), South-East Asian Region (n = 5) and Western Pacific Region (n = 2). In addition, the included studies were from countries with different income groups, with the bulk of studies (16) coming from lower-middle-income countries, 8 from upper-middle-income countries, and 2 from low-income countries. Table II shows all the characteristics of the included studies.

**Table II:** Included studies and their characteristics.

No.	Author and title	Year of publication	Year of study	Study country	Study region (continent)	Main objective/aim of the study	Research design	Study target population	Data source	Type of health provider	Sample size
1	(Le *et al.*, 2018) A cost-effectiveness analysis of freeze-only or fresh embryo transfer in IVF of non-PCOS women	2018	June 2015 and April 2016	Vietnam	Western Pacific Region (WPR)	Examine the cost-effectiveness of a freeze-only versus fresh ET strategy from a patient perspective in the context of a low or middle-income country	Quantitative (cost-effectiveness alongside RCT)	Infertile couples	Medical records	Private	782 couples, 391 couples in each group
2	(Aleyamma *et al.*, 2011) Affordable ART: a different perspective	2011	2008–2010	India	South-East Asian Region (SEAR)	Assessment of low-cost IVF services	Quantitative (experimental study)	Infertile women	Medical records	University hospital	143 women evaluated (104 women underwent embryo transfer)
3	(Sangamithra, 2015) An economic analysis of socio-economic variables and treatment cost of infertility	2015	September 2014 to January 2015	India	South-East Asian Region (SEAR)	To evaluate the relativity between some of socio-economic factors and total cost spent on infertility treatment	Quantitative (cross-sectional)	Infertile women	Infertile women interviews	Private	100 infertile women
4	(Tangwa, 2002) ART and African sociocultural practices: worldview, belief and value systems with particular reference to francophone Africa. In Vayena E. Current practices and controversies in assisted reproduction	2002	1997–2002	Cameroon	African Region (AFR)	Analysis of ART in francophone countries	Qualitative review	Not reported	Not reported	Private and public	1 ART center
5	(Giwa-Osagie, 2002) ART in developing countries with particular reference to sub-Saharan Africa. In Vayena E. Current practices and controversies in assisted reproduction	2002	Not reported	Nigeria Ghana Zimbabwe	African Region (AFR)	The goal was to obtain the following information relating to ART in the subregion: • Is ART being practised? • Where and by whom? • What methods of ART are available? • Cost of ART per cycle. • Statistics and results of ART. • Sources of equipment and consumables. • Technical collaboration, if any. • Assessment of opinion on relevance, need, • affordability and accessibility of ART.	Qualitative	ART centers	Health personnel	Private	Not reported
6	(Huyser and Boyd, 2013) ART in South Africa: The price to pay.	2013	April 2012 to April 2013	South Africa	African Region (AFR)	An overview of cost-drivers within an ART laboratory, such as procedures; sperm preparations; laboratory supplies including embryo culture media and cryopreservation.	Quantitative (cross-sectional study)	ART centers	Health personnel	Private	20 ART units
7	(Gerrits, 2016) Assisted reproductive technologies in Ghana: Transnational undertakings, local practices and ‘more affordable’ IVF	2016	2012 and 2013	Ghana	African Region (AFR)	To provide an insight into the particularities of the uptake of reproductive technologies in Ghana.	Qualitative review	Health professionals and infertile patients	Tariff lists	Private	2 private ART clinics
8	(Abedini *et al.*, 2016) Assisted Reproductive Technology in Iran: The First National Report on Centers, 2011	2016	2011	Iran	Eastern Mediterranean Region (EMR)	First national report on Iranian ART centers. Tracing the accessibility, procedure, cost, and some challenges of IVF in Iran	Quantitative (cross-sectional)	ART sites	Health personnel	Public and private	52 ART units
9	(Dyer and Kruger, 2012) Assisted reproductive technology in South Africa: First results generated from the South African Register of Assisted Reproductive Techniques	2012	2009	South Africa	African Region (AFR)	First report from the South African Register of Assisted Reproductive Techniques.	Quantitative (cross-sectional study)	ART units	Medical records	Public and private	12 Units
10	(Dyer *et al.*, 2013) Catastrophic payment for assisted reproduction techniques with conventional ovarian stimulation in the public health sector of South Africa: frequency and coping strategies.	2013	March 2009 and June 2011	South Africa	African Region (AFR)	How often does out-of-pocket payment (OPP) for assisted reproduction techniques (ART) with conventional ovarian stimulation result in catastrophic expenditure for households?	Quantitative (prospective observational study)	Couples undergoing ART	Post treatment interviews with patients	Public and private	135 couples
11	(Wiersema *et al.*, 2006) Consequences of infertility in developing countries: results of a questionnaire and interview survey in the South of Vietnam. Journal of translational medicine	2006	July until October 2005	Vietnam	Western Pacific Region (WPR)	To explore the psychological, sociocultural, and economic consequences of infertility on couples' life.	Quantitative (cross-sectional study)	Infertile couples	Interviews with patients	Private and public	118 couples (236 participants)
12	(Sangamithra, 2018) Cost incurred and source of finance for the treatment of infertility	2018	Not reported	India	South-East Asian Region (SEAR)	To analyze the principal determinants of total cost incurred for infertility treatment with the help of a multivariate technique	Quantitative (descriptive)	Infertility patients	Interviews with patients	Private	489 respondents
13	(Darvishi *et al.*, 2020) Cost-benefit Analysis of IUI and IVF based on willingness to pay approach; case study: Iran	2020	2016–17	Iran	Eastern Mediterranean Region (EMR)	This study aimed to investigate the value put on IUI and IVF treatments by communities in Iran and the affordability of services based on community preferences.	Quantitative (cost–benefit analysis and cross-sectional)	Couples on fertility treatment	Medical records; cost inquiry from pharmacies and infertility centers.	Private and public	197 IUI medical records and 294 IVF medical records for cost estimation
14	(Ezzatabadi *et al.*, 2016) Determining infertility treatment costs and out of pocket payments imposed on couples	2016	2014	Iran	Eastern Mediterranean Region (EMR)	To determine infertility treatment costs and out of pocket expenditures imposed on couples referred to infertility treatment center in Yazd, Iran	Quantitative (cross-sectional)	Couples who have received IVF	Interviews with patients; medical records	Private	216 couples
15	(Platteau *et al.*, 2008) Four years of IVF/ICSI experience in Kampala (Uganda)	2008	Not reported	Uganda	African Region (AFR)	Analysis of an ART clinic in Uganda	Qualitative review	Not reported	Review (clinic fees)	Private	1 ART center
16	(Hammarberg *et al.*, 2018) Improving access to ART in low-income settings through knowledge transfer: a case study from Zimbabwe	2018	Not reported	Zimbabwe	African Region (AFR)	Describes a model for improving access to ART in low-resource settings.	Qualitative review	Not reported	Review (clinic fees)	Private	1 ART center
17	(Makuch *et al.*, 2011) Inequitable access to assisted reproductive technology for the low-income Brazilian population: a qualitative study.	2011	June 2008 and June 2009	Brazil	Region of the Americas (AMR)	To assess the perspective of health professionals and patients with respect to access to ART procedures within the public health network	Qualitative	Health professionals and ART patients	Interviews	Public	19 health professionals at 5 ART centers in the public sector; 48 patients (men and women)
18	(Inhorn and Gürtin, 2012) Infertility and Assisted Reproduction in the Muslim Middle East: Social, Religious, and Resource Considerations	2012	Not reported	Lebanon Egypt	Eastern Mediterranean Region (EMR)	Discusses the social, religious, and resource considerations around infertility and the provision of assisted reproductive technologies in the Muslim Middle East.	Qualitative review	Review (not reported)	Review (not reported)	Private and public	Review (not reported)
19	(Manzur *et al.*, 2012) Inseminación intrauterina en mayores de 38 años, ¿vale la pena? *Translated:* Intrauterine insemination in over 38 years, is it worth the penalty?	2012	January 2000 and September 2011	Chile	Region of the Americas (AMR)	To determine cost-effectiveness in homologous intrauterine insemination (IUI) outcomes in women 38 years or older in a human reproduction unit and compare results with those of women of similar age treated with complex assisted reproduction technology as published by the Latin American Assisted Reproductive Registry (RedLara) 2009	Quantitative (retrospective, comparative study)	Women over 38 years old treated with homologous intrauterine insemination. interventions:	Medical records	Private	5421 cycles of homologous IUI
20	(Roa-Meggo, 2012) La infertilidad como problema de salud pública en el Perú *Translated*: Infertility as a public health problem in Peru	2012	Not reported	Peru	Region of the Americas (AMR)	An analysis on infertility in Peru	Qualitative review	Review (not reported)	Review (not reported)	Public and private	Review (not reported)
21	(Makuch *et al.*, 2010) Low priority level for infertility services within the public health sector: A Brazilian case study	2010	June 2008 to June 2009	Brazil	Region of the Americas (AMR)	Assessed the availability of public sector infertility services, including assisted reproduction technology (ART), in Brazil.	Quantitative (cross-sectional)	State authorities and ART center management	Interviews	Public	24 authorities from the State Health Secretariats and Federal District. 39 authorities from the Municipal Health Secretariats. 26 directors of the referral centers
22	(Andres, 2019) Necesidad de un marco legal para regular la reproducción humana asistida en el Ecuador *Translated*: Need for a legal framework to regulate assisted human reproduction in Ecuador	2019	2010	Ecuador	Region of the Americas (AMR)	To determine the existing legal vacuum in Ecuador in relation to the regulation of assisted human reproduction methods	Qualitative	Public servants, doctors, lawyers and citizens	Ecuadorian Center for Human Reproduction, 2010	Public and private	50 people
23	(Khalifa, 2012) Reviewing infertility care in Sudan; sociocultural, policy and ethical barriers	2012	September 2011 to November 2011	Sudan	Eastern Mediterranean Region (EMR)	Facility-based review of infertility care in Sudan	Qualitative	ART centers	Interviews of lead physicians	Private	7 ART centers
24	(Widge, 2005) Seeking conception: Experiences of urban Indian women with in vitro fertilization	2005	1997 and 2000	India	South-East Asian Region (SEAR)	Reports on a study of involuntarily childless Indian women/couples seeking *in vitro* fertilization (IVF). The focus is on the social context of infertility and on women's perceptions of and experiences with IVF.	Qualitative	Childless women	Interviews	Private	22 women
25	(Nahar and Richters, 2011) Suffering of childless women in Bangladesh: the intersection of social identities of gender and class	2010	2003–2004	Bangladesh	South-East Asian Region (SEAR)	Addresses the impact of class differences on the gender-related suffering of childless women in the socially very hierarchically structured society of Bangladesh	Qualitative	Childless women	Interviews	All providers (formal and informal-traditional, religious, etc.)	20 rural poor illiterate and 11 urban educated middle class childless women
26	(Gwet-Bell *et al.*, 2018) The 5 main challenges faced in infertility care in Cameroon	2018	Not reported	Cameroon	African Region (AFR)	Summarises the 5 main challenges in infertility care that Cameroonians face	Qualitative review	Review (not reported)	Review (not reported)	Public and private	Review (not reported)

ET, embryo transfer; IUI, intra-uterine insemination; PCOS, polycystic ovary syndrome.

While all studies provided data on the direct medical cost for ART, only three reported direct non-medical costs (Table III). Just under half of the studies (n = 12) reported direct medical costs paid to private healthcare providers, while 9 reported on costs across private and public healthcare providers. Two studies reported costs in public healthcare only, while three studies did not state the health provider type. Eleven studies originated from countries with an ART regulation or law, while 14 were conducted in countries without ART policy. One multi-country study covered countries with (Egypt) and without (Lebanon) ART regulation. Also, 17 studies were conducted in countries with no government financing for ART, while 8 studies were from countries with partial government financing or subsidies (Table III).

**Table III:** Characteristics of the included studies.

Study characteristics	Categories	African Region (AFR)	Eastern Mediterranean Region (EMR)	Region of the Americas (AMR)	South-East Asian Region (SEAR)	Western Pacific Region (WPR)	Total studies	Percent
**Study design**	Quantitative	3	3	2	3	2	13	50.0%
	Qualitative	1	1	2	2	0	6	23.1%
	Reviews	5	1	1	0	0	7	26.9%
**Language**	English	9	5	2	5	2	23	88.5%
	Spanish	0	0	3	0	0	3	11.5%
**Direct** **costs**	Medical costs	9	5	5	5	2	26	100.0%
	Non-medical costs	1	1	0	0	1	3	11.5%
**ART policy/regulation**	Absent policy	6	1*	2	5	0	14	53.8%
	Present policy	3	3*	3	0	2	11	42.3%
**ART financing**	No financing	6	1*	3	5	2	17	65.4%
	Partial/subsidized	3	3*	2	0	0	8	30.8%
**Health provider**	Private and public	4	2	0	2	1	9	34.6%
	Private	5	2	1	3	1	12	46.2%
	Public	0	0	2	0	0	2	7.7%
	Not stated	0	1	2	0	0	3	11.5%
**Countries income groups**	Low income	1	1	0	0	0	2	7.7%
	Lower-middle income	5	4	0	5	2	16	61.5%
	Upper-middle income	3	0	5^†^	0	0	8	30.8%
**Total**	No. of studies	**9**	**5**	**5**	**5**	**2**	**26**	**100.0%**
	Percentage	**35%**	**19%**	**19%**	**19%**	**8%**		

* One multicountry study with Egypt and Lebanon; Egypt has an ART law while Lebanon does not.

† Chile is currently a high-income country, but it was an upper-middle-income country when primary data were collected/published.

### ART costs in LMICs

#### Direct medical costs

Direct medical costs for one ART cycle included payments for diagnosis, procedural costs, laboratory tests, and drugs/medications. Table IV captures the range of direct costs per region, with further details presented in Supplementary Table SIII. The bottom ranges in region of Americas and Asia were based on studies that reported only costs of ovarian stimulation or costs using a minimal stimulation in good prognosis patients and excluding costs related to the staff and institutional overheads, respectively.

**Table IV:** Maximum and minimum ART treatment costs across regions (USD Original costs and PPP adjusted).

	Direct medical cost		Non-medical costs^†^	
	Original costs	Adjusted costs	Original costs	Adjusted costs
WHO Region	(US$)	(US$ PPP)	(US$)	(US $ PPP)
African Region (AFR)	1180.08–4385.13	2295.87–9765.63	17.68	26.87
Eastern Mediterranean Region (EMR)	1000.00–3500.00	3436.43–6770.83	428.86	1168.56
Region of the Americas (AMR)	2000.00–6300.00	3086.42–12 092.13	Not reported	Not reported
South-East Asian Region (SEAR)	1000.00–5596.38	2109.38–18 592.63	Not reported	Not reported
Western Pacific Region (WPR)	1398.06–3000.00	4185.81–12 931.03	756.76	2045.96

† Only three studies from different regions reported non-medical costs.

Private sector care incurred the highest costs [USD18 592.63 in India (Sangamithra, 2018) and USD9 765.63 in Ghana (Giwa-Osagie, 2002)] while, as expected, lower costs were reported for public sector care where ART costs were restricted to payments for medications (Brazil; Makuch *et al.*, 2010, 2011) or subsidized by the institution (South Africa; Dyer and Kruger, 2012). The remaining studies did not distinguish between the two health sectors.

According to the majority of the studies, the highest costs were related to laboratory costs, procedural costs, equipment, and drugs (Khalifa, 2012; Ezzatabadi *et al.*, 2016). For instance, a study in South Africa reported that laboratory services contributed to between 35% and 48% of ART fees paid by patients (Huyser and Boyd, 2013).

#### Direct non-medical costs

Non-medical costs were reported by three studies and ranged from USD26.87–USD2045, as shown in Table IV. The higher costs were related to food, accommodation, and travel for patients living remotely from the treatment institutions. Further details are captured in Supplementary Table SIII.

### Correlation between ART medical costs and annual GDP per capita

**Figure 2.**
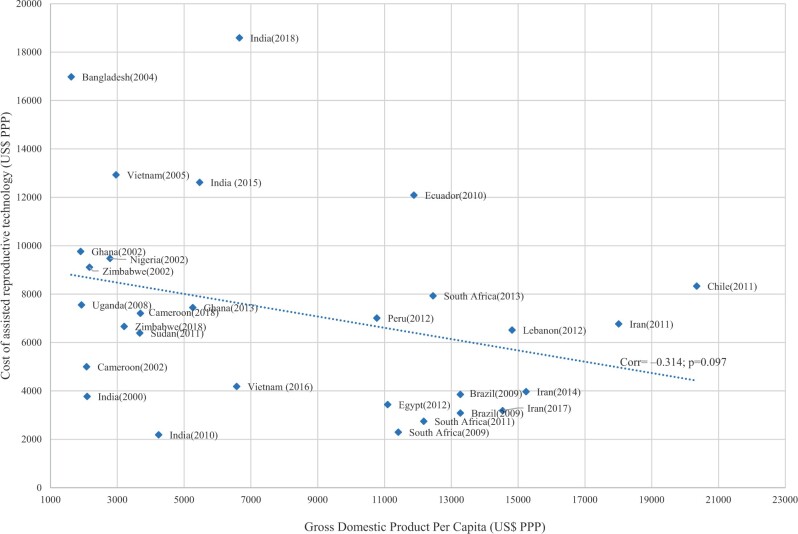
**Direct costs for one ART cycle and GDP per capita.** Cost of ART in US dollars purchasing power parity (USD PPP) versus GDP per capita in USD PPP. A negative correlation between GDP per capita and the cost of one ART cycle is shown (Pearson correlation coefficient = −0.314; *P* = 0.097).

As a measure of affordability, we compared the PPP-adjusted direct medical costs with the annual PPP-adjusted GDP per capita, as shown in Fig. 2. The scatter graph shows a negative correlation, implying that lower GDP per capita was associated with a relatively higher cost for one ART cycle. In contrast, relative costs for one ART cycle in higher GDP countries were lower compared to that in lower GDP per capita countries. The figure also shows a clustering of costs among countries with similar GDP per capita. For instance, lower-middle-income African countries cluster between USD5000 and USD10 000 for one ART cycle, while upper-middle-income countries, such as South Africa, Brazil, and Iran, cluster between USD2000 and USD4000 for one ART cycle.

In more than half of the countries, the direct cost for one ART cycle was higher than the average annual GDP per capita. Specifically, in Africa and South-East Asia, the medical costs were on average 2- to 3-fold higher than the average GDP per capita (227.7% and 327.2%, respectively), while variations ranged between 20.1% of the average GDP per capita in South Africa and 513.1% in Ghana and between 51.6% in India and 1047.9% in Bangladesh, with the latter being the outlier across all countries and regions.

In contrast, in the Eastern Mediterranean region, the costs were an average of 32.1% of the GDP per capita, ranging between 21.9% in Iran and 44% in Lebanon, while in the Americas, the percentage cost ranged between 23.3% in Brazil and 101.8% in Ecuador.

Expressing medical costs as a share of average annual income shows that patients paid significantly higher than their average annual income for one ART cycle in countries with no financing mechanisms, for example, Zimbabwe (456.8%), Sudan (401.5%), Ecuador (243.9%), India (166.4%), and Vietnam (106.3%). In contrast, patients in countries with financing mechanisms such as Iran (54.9%) spent lower than their average annual income on one ART cycle. Conversely, there were nuances in some countries with partial government financing, such as South Africa, where patients who sought infertility treatment in the private sector spent significantly more than their average annual income (194.3%) compared to those who sought infertility treatment in the public sector (56.2%). Supplementary Table SIV summarizes the medical costs as a percentage share of annual GDP per capita and average income.

### ART regulations and financing mechanisms

ART regulations and financing were largely absent in Africa and South-East Asia while more often present in the Americas and the Eastern Mediterranean regions (Table V). Supplementary Table SV presents further detailed information on GDP per capita, ART regulations, and financing per study country.

**Table V:** Studies by the presence of ART regulation and financing mechanisms.

WHO Region	ART regulation/law		ART financing	
	Present	Absent	Present (partial/subsidized)	Absent
African Region (AFR)	3	7	3	7
	South Africa (6,9,10)*	Cameroon (4); Nigeria (5); Ghana (5,7); Zimbabwe (5); Uganda (15)	South Africa (6,9,10)	Cameroon (4); Nigeria (5); Ghana (5,7); Zimbabwe (5); Uganda (15);
Eastern Mediterranean Region (EMR)	3	2	3	2
	Iran (8,13,14); Egypt (18)	Lebanon (18); Sudan (23)	Iran (8,13,14); Egypt (18)	Lebanon (18); Sudan (23)
Region of the Americas (AMR)	3	2	3	2
	Brazil (17, 21); Chile (19)	Peru (20); Ecuador (22)	Brazil (17, 21); Chile (19)	Peru (20); Ecuador (22)
South-East Asian Region (SEAR)	0	5	0	5
	None	India (2,3,12,24), Bangladesh (25)	None	India (2,3,12,24), Bangladesh (25)
Western Pacific Region (WPR)	2	0	0	2
	Vietnam (1, 11)	None	None	Vietnam (1, 11);

*The numbers in brackets refer to the study number ‘No.' in Table II.

## Discussion

This systematic review adds to the evidence on the patients’ cost for ART in LMICs and how these costs compare between countries and regions adjusted for purchasing power parity while concomitantly providing a measure of affordability through the comparison of direct costs with GDP per capita and average annual income.

This review observed that the direct medical costs paid by patients for infertility treatment are often higher than the GDP per capita, making it unaffordable for most people, considering that the income of many people is below the national average. In addition, when using average income as a marker of affordability, patients spent approximately half of their average annual income on one ART cycle in countries with mechanisms for government financing. In contrast, in countries with no mechanisms for financing, the cost for one cycle was even more than double their average annual income. This represents prohibitively high costs for large parts of the populations, even in countries with government financing. The observed negative correlation between direct costs and both GDP per capita and average income has implications for inequity in access to ART. At the same time, patients are at a high risk of incurring catastrophic expenditure because not all those unable to afford it are willing to forgo the treatment due to cost, given the strong desire and social expectations to have a child (Dyer *et al.*, 2013), rendering infertility a ‘medical and social poverty trap’ for many couples in LMICs (Sangamithra, 2015).

In addition to the findings related to affordability, this review shows that in contrast with many, but not all, HICs, ART is generally not financed by governments in LMICs and is largely unregulated and omitted from government policies. The absence of ART policies implies a lack of an appropriate mandate and accountability framework within which provision and financing of ART can safely occur. Therefore, the absence of ART policies potentially plays a role in limiting coverage of infertility treatment by national and private health insurance, thereby sustaining OOP.

Facilitated by the conversion into international dollars (PPP), our review highlights variations in LMICs across the globe and within regions in relation to affordability and emphasizes the link with both public financing and the presence of ART policies. We noted significantly higher costs and lower affordability in Africa and South-East Asia relative to regions of the Americas and the Eastern Mediterranean. These variations emerged due to differences in whether treatment was accessed in the public or private health sector, the level of financing or subsidization, and the presence of ART policies. In spite of these variations, costs were comparatively high, resulting in very low affordability.

This review advances the application of PPP to appraise medical costs when assessing variations across and within LMICs. Furthermore, while other studies (Chambers *et al.*, 2009) have assessed ART cost and its variations in HICs, this review focuses on LMICs. In addition, we highlight the importance of contextualizing affordability based on both GDP per capita and average annual income. Our analysis shows that the proportion of ART costs against average income is higher than the proportion of ART costs against GDP per capita. This points to the role of income distribution on access to ART services. Therefore, in LMICs, taking into account income distribution provides a more realistic estimate of the burden of treatment costs than relying solely on GDP per capita, which could underestimate the impact at the household level. As expected, wealthier countries have better affordability, hence high utilization of ART (Chambers *et al.*, 2014; Lass *et al.*, 2019).

The findings of this review point to several implications. The first is the need for financing and regulatory frameworks in LMICs because these affect pricing and affordability. Many countries in LMICs have ART centers operating without the appropriate regulations and guidelines (Giwa-Osagie, 2002), which has implications for the pricing of services. Although running costs incurred by ART centers are partly due to the complexity of the procedure, they are also strongly influenced by the need for highly skilled staff and sophisticated equipment as well as drugs and consumables, all of which are largely imported into LMICs (Huyser and Boyd, 2013; Gerrits, 2016). Enabling regulatory frameworks would both assist with service provision and containment of pricing. Second, in countries with financial subsidization, there is a need to review the scope of the reimbursements, given that even when partial support is provided to patients, they still spend a considerable amount of money on treatment. Third, future studies should aim to collect more comprehensive data on the direct and indirect costs of ART to better inform costing and financing decisions and to also quantify both direct medical and direct non-medical costs. Indeed, different stakeholders including national and regional ART registries have a role to play in expanding the metrics that they collect to include economic data, in addition to the routinely collected clinical data.

ART financing by the governments in LMICs depends mainly on the cost–benefit value perceived by the state, given the relatively high ART costs (Darvishi *et al.*, 2020). In this regard, financing ART may represent a good governmental investment by enhancing immediate reproductive health while also generating positive financial returns in future tax contributions, including in LMICs (Connolly *et al.*, 2021). Further opportunities to mitigate costs of treatment in low resource settings could include the implementation of low-cost options for ART (Chiware *et al.*, 2021); however, low cost should not lead to a compromise on quality (Aleyamma *et al.*, 2011; Arakkal *et al.*, 2020). Another alternative would be for governments to collaborate with the private sector through public–private partnerships (PPPs) to finance ART. By harnessing the resources of the private sector, states can use public–private engagement as a tool to close funding gaps in healthcare delivery and advance public health goals (Whyle and Olivier, 2016; Babacan, 2021).

The provision of infertility treatment is a complex issue that is compounded by a lack of political will to prioritize infertility, particularly in the context of other health problems such as high rates of maternal morbidity and mortality, unmet needs in contraception, vaccine preventable diseases, and emerging infectious diseases, which are deemed more important. However, infertility is itself a widely prevalent cause of significant health burden for millions of people (Makuch *et al.*, 2010; Mascarenhas *et al.*, 2012; Cox *et al.*, 2022) that should be the addressed alongside other health needs to achieve universal health coverage (Starrs *et al.*, 2018). The results of this review add to the calls for governments in LMICs to increase investments in the provision of fertility treatment, by better integrating infertility in the national health policy and financing (WHO, 2020; Connolly *et al.*, 2021). Yet, in comparison with HICs, this review demonstrates that there are limited data on costs of ART from LMICs to inform policies and financing. Moreover, research on ART affordability is predominated by HICs, despite the absence of reimbursement and lower average incomes in most LMICs. Given the documented importance of costs and levels of reimbursement by governments as well as disposable income in determining the affordability of ART (Connolly *et al.*, 2010; Inhorn and Gürtin, 2012; Abedini *et al.*, 2016), more research is needed on financing of infertility treatment in LMICs.

This review has several limitations. First, despite including 26 studies covering 17 countries, it represents a relatively small sample for LMICs. Second, there were variations across studies on what constituted direct medical costs in that some studies included pre-ART work-up and doctors’ fees, while others only included medications. Thus, some costs could be underestimated. Third, despite our comprehensive search strategy in multiple databases, it is still possible that some papers may have been omitted. Finally, several included studies were not primarily designed to examine the cost of ART and thus lacked comprehensive details of the costs. However, our assessment of the risk of bias showed that most of the studies met the minimum quality requirement and the sensitivity analysis showed that exclusion of those with below minimum quality score did not change the conclusions on the outcome of interest. In addition, although this review analyzed the risk of bias and conducted sensitivity analysis, we did not assess the evidence for factors such as inconsistency, indirectness, and imprecision.

## Conclusion

This review advances the assessment of ART affordability in LMICs, facilitated by both average income and GDP per capita. This review points to the important correlation of ART costs with national and individual wealth impacting on access in LMICs. Our findings show prohibitive costs of ART in LMICs that vary across and within regions, compounded by the absence of ART policies and financing mechanisms. Therefore, it is critical for governments in LMICs to prioritize ART regulations and institute financing mechanisms to improve equity in access to infertility treatment.

## Supplementary Material

hoad007_Supplementary_Data

## Data Availability

All data used for the study have been included in the article and its Supplementary material.
