# Supplementary material for: Financial costs of assisted reproductive technology for patients in low- and middle-income countries: a systematic review
Source: Hum Reprod Open. 2023 Mar 1;2023(2):hoad007. doi: 10.1093/hropen/hoad007 (PMC10029849; doi:10.1093/hropen/hoad007)
Supplement: hoad007_Supplementary_Data [file hoad007_supplementary_data.docx]

**Supplementary Table SI: Search string of PubMed database**

| **Database** | **Search string** |
| --- | --- |
| PubMed (including MEDLINE) | ("developing countries"[MeSH Terms] OR costs[MeSH Terms]) OR "health expenditures"[MeSH Terms] OR fee for service [MeSH Terms] AND "reproductive techniques, assisted"[MeSH Terms] OR "fertilization in vitro"[MeSH Terms] OR "insemination, artificial"[MeSH Terms] OR [LMICs filter ((Cost*[Title/Abstract])) OR ("Out-of-pocket*"[Title/Abstract]) OR (econom*[Title/Abstract]) OR (payment*[Title/Abstract]) OR ("fee for service"[Title/Abstract]) OR (expenditure[Title/Abstract]) OR ("Financial burden"[Title/Abstract]) AND (humans[Filter]) AND ((infertil*[Title/Abstract]) OR (subfertil*[Title/Abstract]) OR (steril*[Title/Abstract]) OR (infecund*[Title/Abstract]) OR (subfecund*[Title/Abstract]) OR (childless*[Title/Abstract]) OR (Barren[Title/Abstract]) OR ("assisted reproductive techn*"[Title/Abstract]) OR (" in vitro fertilisation"[Title/Abstract]) OR ("artificial insemination"[Title/Abstract]) OR ("Intrauterine insemination"[Title/Abstract]) OR ("intracytoplasmic sperm injection"[Title/Abstract]) AND ((humans[Filter]) AND (2001:2020[pdat]))) |

**Supplementary Table SII: ICROMS quality assessment scores of the studies**

|  | ***Study*** | ***Research Design*** | **1. Clear aims and justification** | **2 Managing bias in sampling or between groups** | **3 Managing bias in outcome measurements and Blinding** | **4 Managing bias in follow-up** | **5 Managing bias in other study aspects** | **6 Analytical rigor** | **7 Managing bias in reporting/ethical considerations** | **Total** |
| --- | --- | --- | --- | --- | --- | --- | --- | --- | --- | --- |
| 1 | Le et al., 2018 | Quantitative (cost-effectiveness analysis within a randomized controlled trial (RCT) | 2 | 2 | 5 | 1 | 1 | 1 | 5 | **17** |
| 2 | Aleyamma et al., 2011 | Quantitative (Experimental study) | 3 | 2 | 2 | 1 | 3 | 2 | 9 | **22** |
| 3 | Sangamithra, 2015 | Quantitative  (Cross-sectional study) | 3 | 2 | 2 | 1 | 3 | 2 | 7 | **20** |
| 4 | Tangwa, 2002 | Qualitative Review | 3 | 0 | 0 | 0 | 1 | 1 | 1 | **6** |
| 5 | Giwa-osagie, 2002 | Qualitative | 3 | 1 | 1 | 0 | 1 | 1 | 4 | **11** |
| 6 | Huyser & Boyd, 2013 | Quantitative  (Cross-sectional study) | 3 | 0 | 2 | 0 | 0 | 2 | 8 | **15** |
| 7 | Gerrits, 2016 | Qualitative Review | 4 | 1 | 1 | 0 | 1 | 1 | 1 | **9** |
| 8 | Abedini et al., 2016 | Quantitative  (Cross-sectional) | 4 | 2 | 2 | 1 | 4 | 2 | 9 | **24** |
| 9 | Dyer, S & Kruger, 2012 | Quantitative  (Cross-sectional study) | 4 | 2 | 2 | 1 | 4 | 2 | 9 | **24** |
| 10 | Dyer, S et al., 2013 | Quantitative (Prospective observational study) | 3 | 2 | 2 | 1 | 3 | 2 | 9 | **22** |
| 11 | Wiersema et al., 2006 | Quantitative  (Cross-sectional study) | 3 | 2 | 2 | 1 | 3 | 2 | 9 | **22** |
| 12 | Sangamithra, 2018 | Quantitative (Descriptive) | 3 | 2 | 2 | 1 | 2 | 2 | 8 | **20** |
| 13 | Darvishi et al., 2020 | Quantitative (Cost-Benefit Analysis -Cross-sectional) | 4 | 2 | 2 | 1 | 4 | 2 | 9 | **24** |
| 14 | Ezzatabadi et al., 2016 | Quantitative  (Cross-sectional) | 4 | 2 | 2 | 1 | 4 | 2 | 9 | **24** |
| 15 | Platteau et al., 2008 | Qualitative Review | 3 | 1 | 1 | 0 | 0 | 0 | 0 | **5** |
| 16 | Hammarberg et al., 2018 | Qualitative Review | 3 | 1 | 1 | 0 | 0 | 0 | 0 | **5** |
| 17 | María Y. Makuch et al., 2011 | Qualitative | 6 | 2 | 2 | 0 | 2 | 2 | 5 | **19** |
| 18 | Inhorn & Gürtin, 2012 | Qualitative Review | 6 | 2 | 0 | 0 | 1 | 0 | 0 | **9** |
| 19 | Manzur et al., 2012 | Quantitative (Retrospective, comparative study) | 4 | 2 | 2 | 1 | 4 | 2 | 9 | **24** |
| 20 | Roa-Meggo, 2012 | Qualitative Review | 6 | 2 | 0 | 0 | 1 | 0 | 0 | **9** |
| 21 | Maria Y. Makuch et al., 2010 | Quantitative  (Cross-sectional) | 3 | 2 | 2 | 1 | 2 | 2 | 9 | **21** |
| 22 | Andres, 2019 | Qualitative | 6 | 1 | 2 | 0 | 2 | 1 | 4 | **16** |
| 23 | KhaliFa, 2012 | Qualitative | 5 | 1 | 2 | 0 | 1 | 1 | 4 | **14** |
| 24 | Widge, 2005 | Qualitative | 6 | 2 | 2 | 1 | 2 | 2 | 8 | **23** |
| 25 | Nahar & Richters, 2011 | Qualitative | 6 | 2 | 2 | 0 | 2 | 2 | 8 | **22** |
| 26 | Gwet-Bell et al., 2018 | Qualitative Review | 3 | 0 | 1 | 0 | 1 | 0 | 0 | **5** |

**Supplementary Table SIII: Cost data extracted from the studies**

| ***No.*** | ***Author and Title*** | ***Study Country*** | ***Study region*** | ***Research Design*** | ***Direct Medical Cost as reported)*** | ***Direct Medical Cost*** | | ***Indirect Medical Cost*** | |
| --- | --- | --- | --- | --- | --- | --- | --- | --- | --- |
|  |  |  |  |  |  | *Original USD* | *USD*  *adjusted ppp* | *Original USD* | *USD adjusted ppp* |
| 1 | (Le et al., 2018 | Vietnam | Western Pacific Region (WPR) | Quantitative (Cost-effectiveness alongside RCT) | Freeze only: medical €2138.5 (minus 646.9 pregnancy & delivery) €1491.6  Embryo Transfer: medical €1684.1 (minus 650.8 pregnancy & delivery)  Average €1262.4 | $1,398.06 | $4,185.81 | $756.76 | $2,045.96 |
| 2 | (Aleyamma et al., 2011) | India | South-East Asian Region (SEAR) | Quantitative (Experimental study) | Cost per cycle was US$ 675 for IVF and US$ 725 for an ICSI treatment cycle. | IVF $675  ICSI $725 | IVF $2,109.38  ICSI $2,265.63 | Not Reported | Not Reported |
| 3 | (Sangamithra, 2015) | India | South-East Asian Region (SEAR) | Quantitative (Cross-sectional) | These costs were based on calculated costs from each age category  Average Rs 2,38,000 | $3,709.94 | $12,618.84 | Not Reported | Not Reported |
| 4 | (Tangwa, 2002) | Cameroon | African Region (AFR) | Qualitative Review | 1 million CFA francs (about US$ 1700) today | $1700 | $5,000 | Not Reported | Not Reported |
| 5 | (Giwa-osagie, 2002) | Nigeria  Ghana  Zimbabwe | African Region (AFR) | Qualitative | The cost of one IVF procedure US$ 1200 to US$ 4000  Nigeria -a cycle costs about US$ 2500; a cycle of IVF costs about US$ 2000–US$ 2700  The IVF centre in Zimbabwe charges US$ 2500 per cycle of IVF, or US$ 3500 per cycle including drugs.  Ghana $2500 | Nigeria $2,350  Ghana $2,500 Zimbabwe $3,500 | Nigeria $9,475.80  Ghana $9,765.63 Zimbabwe $9,114.58 | Not Reported | Not Reported |
| 6 | (Huyser & Boyd, 2013) | South Africa | African Region (AFR) | Quantitative (Cross-sectional study) | The average fees per procedure for 20 practices in the private sector in South Africa are: (i) IUI: € 542 ± €159, (ii) IVF: € 3,255 ± € 576 and (iii) ICSI: €3,302 ± € 625. | IVF $4,322.71  ICSI $4,385.13 | IVF$7,873.48  ICSI $7,987.48 | Not Reported | Not Reported |
| 7 | (Gerrits, 2016) | Ghana | African Region (AFR) | Qualitative Review | Treatment costs were around 2500 euros per IVF cycle (without use of donor material or surrogacy). | $3,320.05 | $7,444.06 | Not Reported | Not Reported |
| 8 | (Abedini et al., 2016) | Iran | Eastern Mediterranean Region (EMR) | Quantitative (Cross-sectional) | In 2011, the mean cost of IVF ranged from $2250 to $3600 in government and private centres.  Average $2,925 | $2,925 | $6,770.83 | Not Reported | Not Reported |
| 9 | (Dyer, S & Kruger, 2012) | South Africa | African Region (AFR) | Quantitative (Cross-sectional study) | Out-of-pocket payment for a standard IVF cycle range from approximately R10 000 (subsidized care in the public sector) to R35 000 (private sector care)  Average R 22500 | $1,180.08 | $2,295.87 | Not Reported | Not Reported |
| 10 | (Dyer, S et al., 2013) | South Africa | African Region (AFR) | Quantitative (Prospective observational study) | Average direct cost (including visit fee, medication cost, laboratory costs and transport to facility) of the current cycle was €1311 Minus transport €12.7 | $1,808.22 | $2,748.05 | $17.68 | $26.87 |
| 11 | (Wiersema et al., 2006) | Vietnam | Western Pacific Region (WPR) | Quantitative (Cross-sectional study) | Total cost for IVF treatment is roughly USD 3.000 | $3,000 | $12,931.03 | Not Reported | Not Reported |
| 12 | (Sangamithra, 2018) | India | South-East Asian Region (SEAR) | Quantitative (Descriptive) | Rs. 1,44,184/- Low income  Rs. 1,35,026/- - Backward communities  95,590/- Scheduled Castes and Rs. 1,03,521- Forward communities  Average Rs 3,82,731 | $5,596.38 | $18,592.63 | Not Reported | Not Reported |
| 13 | (Darvishi et al., 2020) | Iran | Eastern Mediterranean Region (EMR) | Quantitative (Cost-Benefit Analysis  -Cross-sectional) | IUI 19561140 IRR and  IVF 60897610 IRR | $1,832.81 | $4,823.18 | Not Reported | Not Reported |
| 14 | (Ezzatabadi et al., 2016) | Iran | Eastern Mediterranean Region (EMR) | Quantitative (Cross-sectional) | Direct costs: 378.123.54 rial  Out of pocket payments 33.032.872 rial | $1,457.59 | $3,971.64 | $428.86 | $1168.56 |
| 15 | (Platteau et al., 2008) | Uganda | African Region (AFR) | Qualitative Review | A standard IVF/ICSI treatment with medication costs from $3000 onwards at our clinic) | $3,000 | $7,556.68 | Not Reported | Not Reported |
| 16 | (Hammarberg et al., 2018) | Zimbabwe | African Region (AFR) | Qualitative Review | USD 3500, which covers hospital stay, medication, and gynaecologist and embryologist fees. | $3,500 | $6,666.67 | Not Reported | Not Reported |
| 17 | (Makuch et al., 2011) | Brazil | Region of the Americas (AMR) | Qualitative | US$2000 per IVF/ICSI cycle only for the medication, reaching US$3000 per cycle in the cases when the additional fee was added  Average $2500 | $2,500 | $3,858.02 | Not Reported | Not Reported |
| 18 | (Inhorn & Gürtin, 2012) | Egypt Lebanon | Eastern Mediterranean Region (EMR) | Qualitative Review | The average cost of an ART cycle in the Middle East ranges from the low of about $US 1,000 - Egypt; to the high of about $US 6,000 - UAE), many countries in between $2,000-$5,000 Lebanon - Average $3500 | Egypt $1,000  Lebanon $3,500 | Egypt $3,436.43  Lebanon $6,517.69 | Not Reported | Not Reported |
| 19 | Manzur et al., 2012) | Chile | Region of the Americas (AMR) | Quantitative (Retrospective, comparative study) | IVF - US $ 6,000 | $6,000 | $8,333.33 | Not Reported | Not Reported |
| 20 | (Roa-Meggo, 2012) | Peru | Region of the Americas (AMR) | Qualitative Review | (IVF) treatment in Lima can cost approximately between US $ 3,500 and US $ 5,000  Average $4250 | $4,250 | $7,013 | Not Reported | Not Reported |
| 21 | (Makuch et al., 2010) | Brazil | Region of the Americas (AMR) | Quantitative (Cross-sectional) | Cost for drugs: US$2000 per cycle for IVF or ICSI procedures. | $2,000 (Drugs only) | $3,086.42 | Not Reported | Not Reported |
| 22 | (Andres, 2019) | Ecuador | Region of the Americas (AMR) | Qualitative | Artificial insemination: between $ 200 to $ 300 dollars approximately (Average $250)  Heterologous Artificial insemination - $500  Medication $200-$300  Ultrasound $120  Total $820 - $920 = Average $870  IVF/ICSI  $ 4000 to $ 5000 dollars Plus $1800  Total $5800 to $6800 =$6300 | $6,300 | $12,092.13 | Not Reported | Not Reported |
| 23 | (Khalifa, 2012) | Sudan | Eastern Mediterranean Region (EMR) | Qualitative | Cost of one cycle of IVF/ICSI treatment with drugs starts at 2,500 USD in all centres | $2,500 | $6,393.86 | Not Reported | Not Reported |
| 24 | (Widge, 2005) | India | South-East Asian Region (SEAR) | Qualitative | IVF average cost USD 600 to USD 1000  Average $800 | $800 | $3,773.58 | Not Reported | Not Reported |
| 25 | (Nahar & Richters, 2011) | Bangladesh | South-East Asian Region (SEAR) | Qualitative | Urban women respondents in this study underwent high-tech fertility treatment, which is extremely expensive, ranging from €2000 to €6000  Average € 4000 | $4,975.12 | $16,979.95 | Not Reported | Not Reported |
| 26 | (Gwet-Bell et al., 2018) | Cameroon | African Region (AFR) | Qualitative Review | An IVF cycle, including drugs, costs US$3000. | $3,000 | $7,211.54 | Not Reported | Not Reported |

**Supplementary Table SIV: Proportion of medical costs as a share of GDP per capita and mean income**

| ***WHO Regions*** | ***Study*** | ***Country*** | ***Cost of ART (USD PPP)*** | ***GDP per capital(USD PPP)*** | ***% Cost of treatment/GDP per capita*** | ***Year mean income was derived*** | ***Mean annual Income $PPP*** | ***Cost/Annual income*** |
| --- | --- | --- | --- | --- | --- | --- | --- | --- |
| **African Region (AFR)** | (Tangwa, 2002) | Cameroon | 5000 | 2079.8 | 240.4% | 2014 | 1841.76 | 271.5%† |
|  | (Giwa-osagie, 2002) | Nigeria | 9475.8 | 2780.8 | 340.8% | 2018 | 1021.56 | 927.6%† |
|  | (Giwa-osagie, 2002) | Ghana | 9765.6 | 1903.1 | 513.1% | 2016 | 2473.08 | 394.9%† |
|  | (Giwa-osagie, 2002) | Zimbabwe | 9114.6 | 2167 | 420.6% | 2019 | 1459.32 | 624.6%† |
|  | (Huyser & Boyd, 2013) | South Africa | 7930.5 | 12 450.5 | 63.7% | 2014 | 4081.8 | 194.3%‡ |
|  | (Gerrits, 2016) | Ghana | 7444.1 | 5259.6 | 141.5% | 2016 | 2473.08 | 301.0%† |
|  | (Silke J. Dyer & Kruger, 2012) | South Africa | 2295.9 | 11 409.6 | 20.1% | 2014 | 4081.8 | 56.2%‡ |
|  | (Silke J. Dyer et al., 2013) | South Africa | 2748.1 | 12 172.3 | 22.6% | 2014 | 4081.8 | 67.3%‡ |
|  | (Platteau et al., 2008) | Uganda | 7556.7 | 1926.9 | 392.2% | 2016 | 1169.16 | 646.3%† |
|  | (Hammarberg et al., 2018) | Zimbabwe | 6666.7 | 3206.3 | 207.9% | 2019 | 1459.32 | 456.8%‡ |
|  | (Gwet-Bell et al., 2018) | Cameroon | 7211.5 | 3691.3 | 195.4% | 2014 | 1841.76 | 391.6%† |
| **South-East Asian Region (SEAR)** | (Aleyamma et al., 2011) | India | 2187.5 | 4236.7 | 51.6% | 2011 | 1314.72 | 166.4%‡ |
|  | (Sangamithra, 2015) | India | 12 618.8 | 5464.9 | 230.9% | 2011 | 1314.72 | 959.8%† |
|  | (Sangamithra, 2018) | India | 18 592.6 | 6655.1 | 279.4% | 2011 | 1314.72 | 1414.2%† |
|  | (Widge, 2005) | India | 3773.6 | 2095.6 | 180.1% | 2011 | 1314.72 | 287.0%† |
|  | (Nahar & Richters, 2011) | Bangladesh | 16 980 | 1620.4 | 1047.9% | 2016 | 1419.12 | 1196.5%† |
| **Western Pacific (WPR)** | (Le et al., 2018) | Vietnam | 4185.8 | 6573.1 | 63.7% | 2018 | 3936 | 106.3%‡ |
|  | (Wiersema et al., 2006) | Vietnam | 12 931 | 2960.6 | 436.8% | 2018 | 3936 | 328.5%† |
| **Eastern Mediterranean Region (EMR)** | (KhaliFa, 2012) | Sudan | 6393.9 | 3673.5 | 174.1% | 2014 | 1592.4 | 401.5%‡ |
|  | (Abedini et al., 2016) | Iran | 6770.8 | 18 008.8 | 37.6% | 2018 | 5798.4 | 116.8%† |
|  | (Darvishi et al., 2020) | Iran | 3186.2 | 14 535.9 | 21.9% | 2018 | 5798.4 | 54.9%‡ |
|  | (Ezzatabadi et al., 2016) | Iran | 3971.6 | 15 235.7 | 26.1% | 2018 | 5798.4 | 68.5%† |
|  | (Inhorn & Gürtin, 2012) | Egypt | 3436.4 | 11 092.7 | 31.0% | 2017 | 1869.84 | 183.8%† |
|  | (Inhorn & Gürtin, 2012) | Lebanon | 6517.7 | 14 816.9 | 44.0% | 2011 | 9091.44 | 71.7%‡ |
| **Region of the Americas (AMR)** | (María Y. Makuch et al., 2011) | Brazil | 3858 | 13 269.1 | 29.1% | 2019 | 6720.48 | 57.4%† |
|  | Manzur et al., 2012) | Chile | 8333.3 | 20 342.6 | 41.0% | 2017 | 9304.68 | 89.6%† |
|  | (Roa-Meggo, 2012) | Peru | 7013 | 10 767.7 | 65.1% | 2019 | 4987.56 | 140.6%† |
|  | (Maria Y. Makuch et al., 2010) | Brazil | 3086.4 | 13 269.1 | 23.3% | 2019 | 6720.48 | 45.9%† |
|  | (Andres, 2019) | Ecuador | 12 092.1 | 11 878.7 | 101.8% | 2019 | 4957.08 | 243.9%‡ |

*‡ Medical costs correspond to the year mean income was computed; † Mean income for the year the study was conducted was not available*

**Supplementary Table SV: GDP per capita, ART regulations and financing per study country**

|  | ***Author and Title*** | ***Year of study*** | ***Study Country*** | ***Study region*** | ***GDP per capita in US$ (Year of study)*** | ***GDP per capita (US$ PPP)*** | ***ART regulations*** | ***Financing for ART/infertility treatment?*** |
| --- | --- | --- | --- | --- | --- | --- | --- | --- |
| 1 | (Le et al., 2018 | June 2015 and April 2016 | Vietnam | Western Pacific Region (WPR) | 2192.225 (2016) | 6573.096 | Present | No public insurance coverage/funding/ reimbursement |
| 2 | (Aleyamma et al., 2011) | 2008 to 2010 | India | South-East Asian Region (SEAR) | 1357.564(2010) | 4236.744 | Absent | No public insurance coverage/funding/ reimbursement |
| 3 | (Sangamithra, 2015) | September 2014 to January 2015 | India | South-East Asian Region (SEAR) | 1605.605(2015) | 5464.859 | Absent | No public insurance coverage/funding/ reimbursement |
| 4 | (Tangwa, 2002) | 1997-2002 | Cameroon | African Region (AFR) | 707.888(2002) | 2079.76 | Absent | No public insurance coverage/funding/ reimbursement |
| 5 | (Giwa-osagie, 2002) | Not reported | Nigeria  Ghana  Zimbabwe | African Region (AFR) | Nigeria 741.748 (2002)  Ghana 304.565 (2002)  Zimbabwe 530.531(2002) | Nigeria 2780.792  Ghana 1903.146 Zimbabwe 2167.02 | Absent | No public insurance coverage/funding/ reimbursement |
| 6 | (Huyser & Boyd, 2013) | April 2012 - April 2013 | South Africa | African Region (AFR) | 6832.457(2013) | 12 450.498 | Present | Subsidized in Public sector |
| 7 | (Gerrits, 2016) | 2012 & 2013 | Ghana | African Region (AFR) | 2345.393(2013) | 5259.618 | Absent | No public insurance coverage/funding/ reimbursement |
| 8 | (Abedini et al., 2016) | 2011 | Iran | Eastern Mediterranean Region (EMR) | 7781.406(2011) | 18 008.803 | Present | Partial government support package |
| 9 | (Dyer, S & Kruger, 2012) | 2009 | South Africa | African Region (AFR) | 5862.797 (2009) | 11 409.572 | Present | Subsidized in Public sector |
| 10 | (Dyer, S et al., 2013) | March 2009 and June 2011 | South Africa | African Region (AFR) | 8007.413(2011) | 12 172.315 | Present | Subsidized in Public sector |
| 11 | (Wiersema et al., 2006) | July until October 2005 | Vietnam | Western Pacific Region (WPR) | 687.48(2005) | 2960.641 | Present | No public insurance coverage/funding/ reimbursement |
| 12 | (Sangamithra, 2018) | Not reported | India | South-East Asian Region (SEAR) | 2005.863(2018) | 6655.07 | Absent | No public insurance coverage/funding/ reimbursement |
| 13 | (Darvishi et al., 2020) | 2016–17 | Iran | Eastern Mediterranean Region (EMR) | 5520.31(2017) | 14 535.862 | Present | Partial government financing to clinics |
| 14 | (Ezzatabadi et al., 2016) | 2014 | Iran | Eastern Mediterranean Region (EMR) | 5585.527(2014) | 15 235.705 | Present | Partial government financing to clinics |
| 15 | (Platteau et al., 2008) | Not reported | Uganda | African Region (AFR) | 474.518(2008) | 1926.908 | Absent | No public insurance coverage/funding/ reimbursement |
| 16 | (Hammarberg et al., 2018) | Not reported | Zimbabwe | African Region (AFR) | 1683.741(2018) | 3206.277 | Absent | No public insurance coverage/funding/ reimbursement |
| 17 | (Makuch et al., 2011) | June 2008 and June 2009 | Brazil | Region of the Americas (AMR) | 8597.915(2009) | 13 269.119 | Present | Partial government financing |
| 18 | (Inhorn & Gürtin, 2012) | Not reported | Lebanon  Egypt | Eastern Mediterranean Region (EMR) | Egypt: 3,229.686 (2012)  Lebanon:7950.695(2012) | Egypt:  11 092.743  Lebanon:  14 816.878 | Egypt - Present**  Lebanon - Absent | Egypt: Partial government financing to clinics  Lebanon: No public insurance coverage/funding/ reimbursement |
| 19 | Manzur et al., 2012) | January 2000 and September 2011 | Chile | Region of the Americas (AMR) | 14 637.24(2011) | 20 342.571 | Present | Partial Reimbursement by state |
| 20 | (Roa-Meggo, 2012) | Not reported | Peru | Region of the Americas (AMR) | 6528.972(2012) | 10 767.678 | Absent | No public insurance coverage/funding/ reimbursement |
| 21 | (Makuch et al., 2010) | June 2008 to June 2009 | Brazil | Region of the Americas (AMR) | 8597.915(2009) | 13 269.119 | Present | Partial government financing |
| 22 | (Andres, 2019) | 2010 | Ecuador | Region of the Americas (AMR) | 6183.8 (2019) | 11 878.718 | Absent | No public insurance coverage/funding/ reimbursement |
| 23 | (Khalifa, 2012) | September 2011 to November 2011 | Sudan | Eastern Mediterranean Region (EMR) | 1437.773(2011) | 3673.487 | Absent | No public insurance coverage/funding/ reimbursement |
| 24 | (Widge, 2005) | 1997 and 2000 | India | South-East Asian Region (SEAR) | 443.314 (2000) | 2095.647 | Absent | No public insurance coverage/funding/ reimbursement |
| 25 | (Nahar & Richters, 2011) | 2003–2004 | Bangladesh | South-East Asian Region (SEAR) | 475.292(2004) | 1620.425 | Absent | No public insurance coverage/funding/ reimbursement |
| 26 | (Gwet-Bell et al., 2018) | Not reported | Cameroon | African Region (AFR) | 1534.494(2018) | 3691.334 | Absent | No public insurance coverage/funding/ reimbursement |

**Supplementary Table SV: GDP per capita, ART regulations and financing per study country**

|  | ***Author and Title*** | ***Year of study*** | ***Study Country*** | ***Study region*** | ***GDP per capita in US$ (Year of study)*** | ***GDP per capita (US$ PPP)*** | ***ART regulations*** | ***Financing for ART/infertility treatment?*** |
| --- | --- | --- | --- | --- | --- | --- | --- | --- |
| 1 | (Le et al., 2018 | June 2015 and April 2016 | Vietnam | Western Pacific Region (WPR) | 2192.225 (2016) | 6573.096 | Present | No public insurance coverage/funding/ reimbursement |
| 2 | (Aleyamma et al., 2011) | 2008 to 2010 | India | South-East Asian Region (SEAR) | 1357.564(2010) | 4236.744 | Absent | No public insurance coverage/funding/ reimbursement |
| 3 | (Sangamithra, 2015) | September 2014 to January 2015 | India | South-East Asian Region (SEAR) | 1605.605(2015) | 5464.859 | Absent | No public insurance coverage/funding/ reimbursement |
| 4 | (Tangwa, 2002) | 1997-2002 | Cameroon | African Region (AFR) | 707.888(2002) | 2079.76 | Absent | No public insurance coverage/funding/ reimbursement |
| 5 | (Giwa-osagie, 2002) | Not reported | Nigeria  Ghana  Zimbabwe | African Region (AFR) | Nigeria 741.748 (2002)  Ghana 304.565 (2002)  Zimbabwe 530.531(2002) | Nigeria 2780.792  Ghana 1903.146 Zimbabwe 2167.02 | Absent | No public insurance coverage/funding/ reimbursement |
| 6 | (Huyser & Boyd, 2013) | April 2012 - April 2013 | South Africa | African Region (AFR) | 6832.457(2013) | 12 450.498 | Present | Subsidized in Public sector |
| 7 | (Gerrits, 2016) | 2012 & 2013 | Ghana | African Region (AFR) | 2345.393(2013) | 5259.618 | Absent | No public insurance coverage/funding/ reimbursement |
| 8 | (Abedini et al., 2016) | 2011 | Iran | Eastern Mediterranean Region (EMR) | 7781.406(2011) | 18 008.803 | Present | Partial government support package |
| 9 | (Dyer, S & Kruger, 2012) | 2009 | South Africa | African Region (AFR) | 5862.797 (2009) | 11 409.572 | Present | Subsidized in Public sector |
| 10 | (Dyer, S et al., 2013) | March 2009 and June 2011 | South Africa | African Region (AFR) | 8007.413(2011) | 12 172.315 | Present | Subsidized in Public sector |
| 11 | (Wiersema et al., 2006) | July until October 2005 | Vietnam | Western Pacific Region (WPR) | 687.48(2005) | 2960.641 | Present | No public insurance coverage/funding/ reimbursement |
| 12 | (Sangamithra, 2018) | Not reported | India | South-East Asian Region (SEAR) | 2005.863(2018) | 6655.07 | Absent | No public insurance coverage/funding/ reimbursement |
| 13 | (Darvishi et al., 2020) | 2016–17 | Iran | Eastern Mediterranean Region (EMR) | 5520.31(2017) | 14 535.862 | Present | Partial government financing to clinics |
| 14 | (Ezzatabadi et al., 2016) | 2014 | Iran | Eastern Mediterranean Region (EMR) | 5585.527(2014) | 15 235.705 | Present | Partial government financing to clinics |
| 15 | (Platteau et al., 2008) | Not reported | Uganda | African Region (AFR) | 474.518(2008) | 1926.908 | Absent | No public insurance coverage/funding/ reimbursement |
| 16 | (Hammarberg et al., 2018) | Not reported | Zimbabwe | African Region (AFR) | 1683.741(2018) | 3206.277 | Absent | No public insurance coverage/funding/ reimbursement |
| 17 | (Makuch et al., 2011) | June 2008 and June 2009 | Brazil | Region of the Americas (AMR) | 8597.915(2009) | 13 269.119 | Present | Partial government financing |
| 18 | (Inhorn & Gürtin, 2012) | Not reported | Lebanon  Egypt | Eastern Mediterranean Region (EMR) | Egypt: 3,229.686 (2012)  Lebanon:7950.695(2012) | Egypt:  11 092.743  Lebanon:  14 816.878 | Egypt - Present**  Lebanon - Absent | Egypt: Partial government financing to clinics  Lebanon: No public insurance coverage/funding/ reimbursement |
| 19 | Manzur et al., 2012) | January 2000 and September 2011 | Chile | Region of the Americas (AMR) | 14 637.24(2011) | 20 342.571 | Present | Partial Reimbursement by state |
| 20 | (Roa-Meggo, 2012) | Not reported | Peru | Region of the Americas (AMR) | 6528.972(2012) | 10 767.678 | Absent | No public insurance coverage/funding/ reimbursement |
| 21 | (Makuch et al., 2010) | June 2008 to June 2009 | Brazil | Region of the Americas (AMR) | 8597.915(2009) | 13 269.119 | Present | Partial government financing |
| 22 | (Andres, 2019) | 2010 | Ecuador | Region of the Americas (AMR) | 6183.8 (2019) | 11 878.718 | Absent | No public insurance coverage/funding/ reimbursement |
| 23 | (Khalifa, 2012) | September 2011 to November 2011 | Sudan | Eastern Mediterranean Region (EMR) | 1437.773(2011) | 3673.487 | Absent | No public insurance coverage/funding/ reimbursement |
| 24 | (Widge, 2005) | 1997 and 2000 | India | South-East Asian Region (SEAR) | 443.314 (2000) | 2095.647 | Absent | No public insurance coverage/funding/ reimbursement |
| 25 | (Nahar & Richters, 2011) | 2003–2004 | Bangladesh | South-East Asian Region (SEAR) | 475.292(2004) | 1620.425 | Absent | No public insurance coverage/funding/ reimbursement |
| 26 | (Gwet-Bell et al., 2018) | Not reported | Cameroon | African Region (AFR) | 1534.494(2018) | 3691.334 | Absent | No public insurance coverage/funding/ reimbursement |
